# Construction of immune-related and prognostic lncRNA clusters and identification of their immune and genomic alterations characteristics in lung adenocarcinoma samples

**DOI:** 10.18632/aging.103251

**Published:** 2020-05-23

**Authors:** Jia Li, Chenyue Zhang, Chenxing Zhang, Haiyong Wang

**Affiliations:** 1 Department of Integrated Chinese and Western Medicine, Affiliated Cancer Hospital of Zhengzhou University and Henan Cancer Hospital, Zhengzhou 450008, China; 2 Department of Integrated Therapy, Fudan University Shanghai Cancer Center, Shanghai Medical College, Shanghai 200032, China; 3 Department of Nephrology, Shanghai Children's Medical Center, Shanghai Jiao Tong University School of Medicine, Shanghai 200127, China; 4 Department of internal Medicine-Oncology, Shandong Cancer Hospital and Institute, Shandong First Medical University and Shandong Academy of Medical Sciences, Jinan 250117, China

**Keywords:** immune-related lncRNA, lung adenocarcinoma, immune cells infiltration, genomic alteration, cluster

## Abstract

Long non-coding RNAs (lncRNAs) play an important role in various biological processes of lung adenocarcinoma (LUAD), such as immune response regulation, tumor microenvironment remodeling and genomic alteration. Nevertheless, immune-related lncRNAs and their immune and genomic alterations characteristics in LUAD samples still remain unreported. Here, using various public databases, statistic and software tools, we constructed two immune-related lncRNA clusters with different immune and genomic alterations characteristics. Notably, cluster 1 had a stronger immunosuppressive tumor microenvironment (TME) and a higher mutation frequency than cluster 2, especially the mutant genes, such as Kelch-like ECH-associated protein 1 (KEAP1) and toll like receptor 4 (TLR4). In cluster 1, both the amplified and deleted portions of copy number variation (CNV) segments were enriched and cyclin dependent kinase inhibitor 2A (CDKN2A) was significantly deleted. GSVA analysis revealed that these immune-related lncRNAs may be involved in stem cell and EMT functions. Furthermore, cluster 1 was related to worse prognosis of LUAD patients. Therefore, we constructed two immune-related and prognostic lncRNA clusters and identified their immune and genomic alterations characteristics in LUAD samples, which could well divide LUAD patients into different immune phenotypes and help to understand immune molecular mechanisms of LUAD.

## INTRODUCTION

Lung adenocarcinoma (LUAD) is the most prevalent pathological subtype of non small cell lung cancer (NSCLC), which accounts for approximately 40% of lung cancer worldwide [1, 2]. The average 5-year survival rate of patients with LUAD is only 18%, although comprehensive treatments such as surgery and targeted therapies have improved clinical therapies [3]. Recently, immunotherapy strategies have exhibited an unexpected antitumor effect in LUAD [4, 5]. However, fewer patients respond to this therapy, and there is no clearly molecular stratification of the patients [6, 7]. Thus, deeply understanding of immune molecular mechanisms and underlying subtypes of LUAD is of great significance for more effective treatment options.

Long non-coding RNAs (lncRNAs) are non-coding RNAs without protein-coding capacity and are >200 nucleotides (nt) long [8]. LncRNA functions have been discovered in chromatin interactions, transcriptional regulation, RNA processing, mRNA stability or translation, and signal cascade regulation [9–14]. Increasing evidence shows that lncRNAs can regulate not only the innate immune response but also the more sophisticated adaptive immune response as well as immune cell development [15–18]. Moreover, lncRNAs may be pivotal regulators in remodeling the tumor microenvironment (TME) [17, 19, 20], which forms complex and heterogeneous environments consisting of multiple cells, such as infiltrating immune cells and stromal cells [21]. For instance, lnc-EGFR also acts as an immune-suppressor by promoting regulatory T cells differentiation in hepatocellular carcinoma [22]. NF-κB interacting NKILA (an lncRNA) enhances T cell sensitivity to activation-induced cell death by mechanically inhibiting NF-κB signaling [23]. Lymph node metastasis associated transcript 1 (LNMAT1), also a new lncRNA, is involved in the regulation of C-C motif chemokine ligand 2 (CCL2) recruiting macrophages into the tumor [24]. The involvement of lncRNAs in immune regulation is complicated, and many key immune regulatory lncRNAs have not yet been identified. Hence, it is urgent needed to identify new immune-related lncRNAs and elucidate their interactions with immune system in TME.

The development and progression of cancer involve various types of genomic alterations, including somatic mutations, copy number variation (CNV), and other changes in gene expression [25]. Somatic mutations are considered the initiator of cancer by altering genetic and epigenetic mechanisms [26]. CNVs can cause heterogeneity of genomic and molecular phenotypes, leading to the occurrence and development of complex diseases including cancer [27, 28]. Multidimensional genomics data provide more extensive insights into the genomic alterations affected by lncRNAs through various effects [29, 30]. For instance, the prognostic in lung adenocarcinoma LncRNA1 (PILAR1), a new prognostic lncRNA, is associated with a high mutation rate of Kelch-like ECH-associated protein 1(KEAP1) [31]. LOC101927151, LINC00861 and LEMD1-AS1 are LncRNAs with transcriptional dysregulation caused by CNV abnormality and are prognostic biomarkers in ovarian cancer [28]. Although several lncRNAs have been shown to be involved in genomic alterations, the relationship between immune-related lncRNA and genomic alterations in LUAD still remain unreported.

In this study, we firstly generated the co-expression network of the immune-related mRNAs and lncRNAs to obtain 147 immune-related and prognostic lncRNAs. Then we constructed two immune-related lncRNA clusters in LUAD samples. Further, we analyzed the characteristics of immune microenvironment and genomic alterations, including somatic mutations, CNVs in different immune-related lncRNA clusters. Finally, we investigated whether immune-related lncRNA clusters could predict the prognosis of the patients.

## RESULTS

### Identification of immune-related lncRNAs and mRNAs

**Figure 1:**
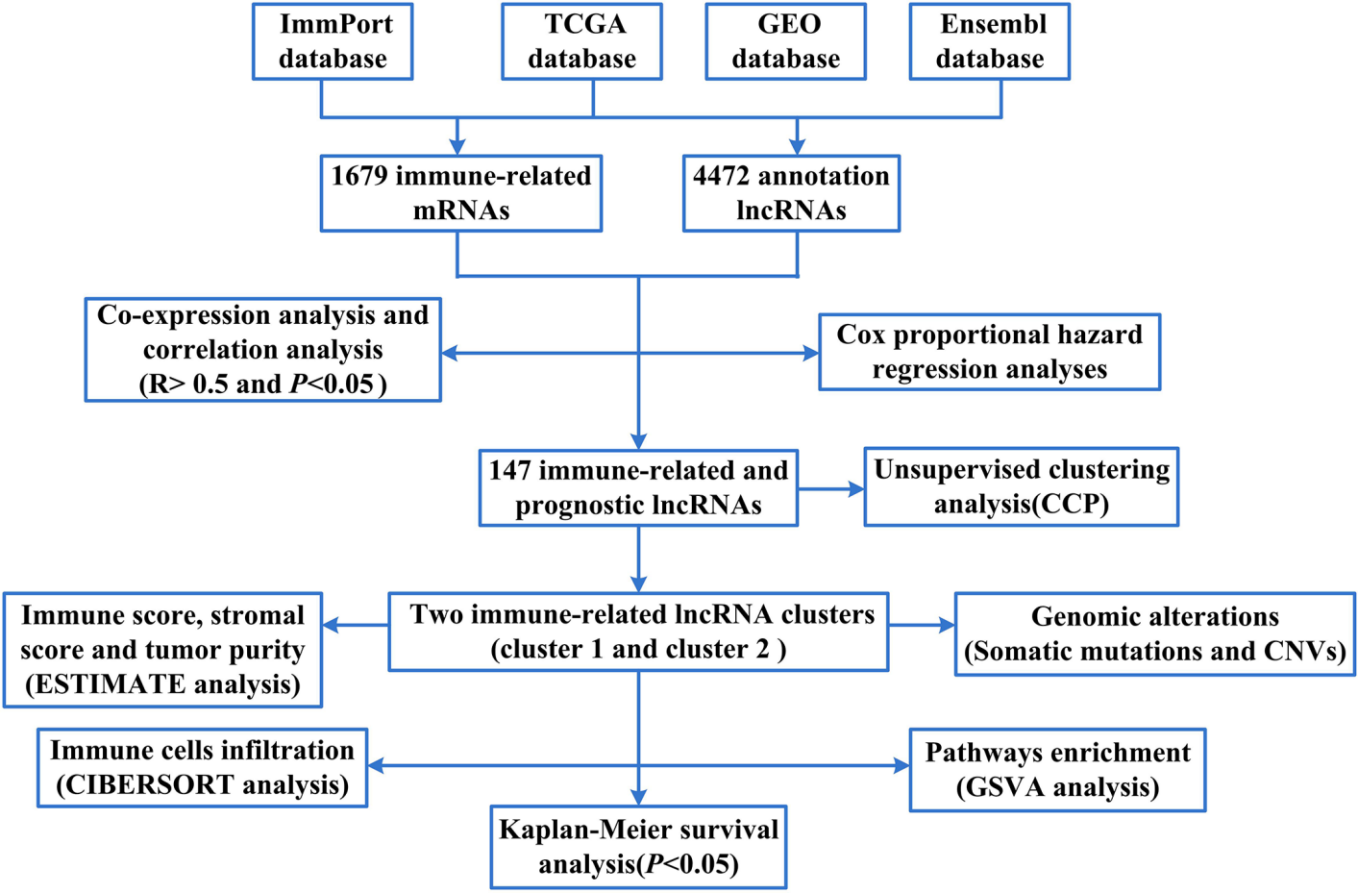
**The flow chart of this study.**

To screen for immune-related mRNAs, we firstly compared the mRNA data of TCGA database and ImmPort database and took the intersection to obtain the immune-related mRNA (1679 immune-related mRNAs). Later, we compared the lncRNAs data of TCGA and GEO database with Ensembl database and took the intersection. Thus, about 4472 annotation lncRNAs were obtained. Finally, we sorted lncRNAs according to the number of co-expressed lncRNAs and immune-related mRNAs (degree) to obtain immune-related lncRNAs (Supplementary Table 1). The flow chart of this study was shown in Figure 1.

**Figure 2:**
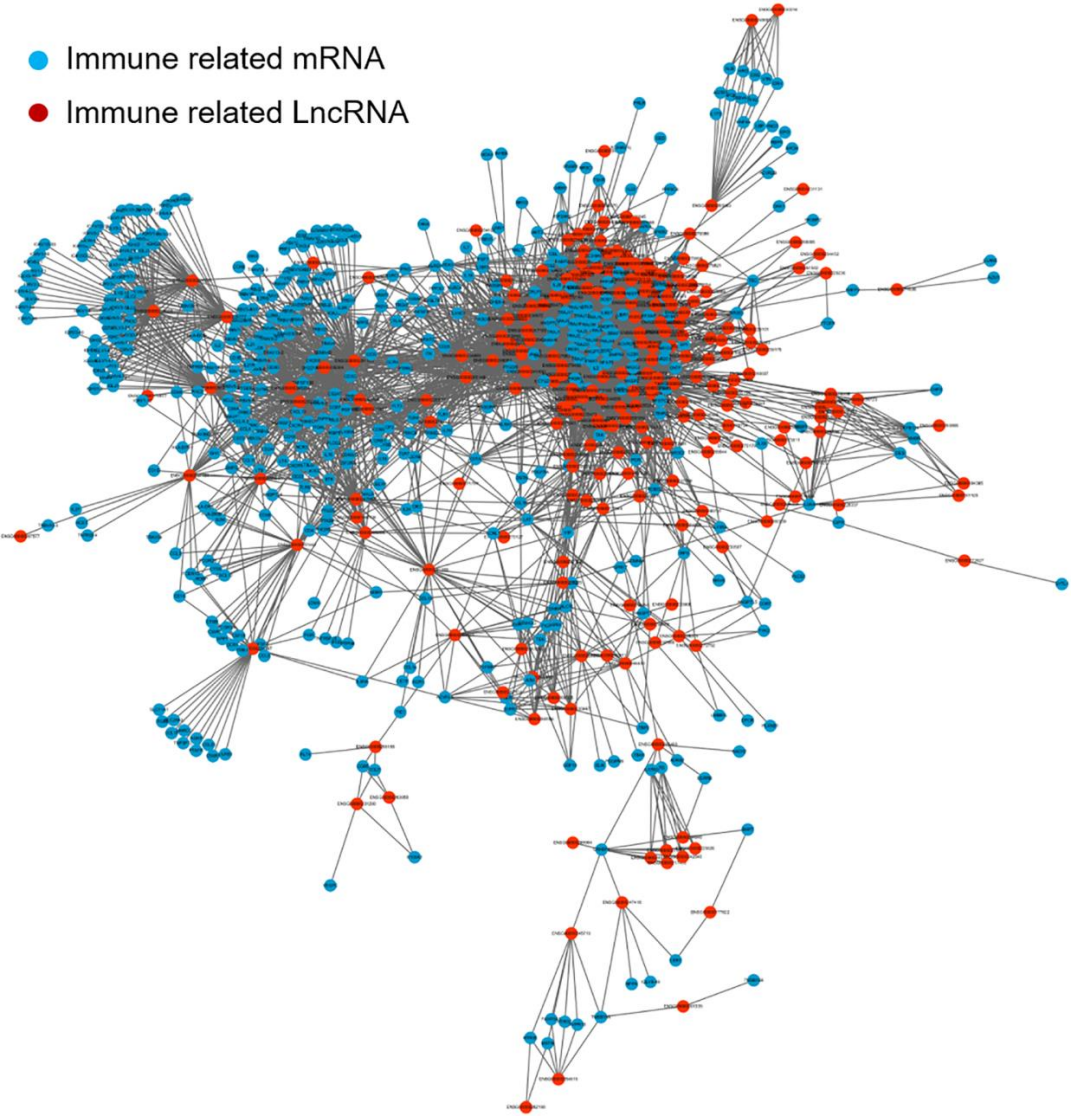
**The co-expression network map between immune-related mRNAs and lncRNAs in LUAD.** Blue represents immune-related mRNA and red represents co-expressed lncRNA. lncRNA: long non-coding RNA; LUAD: lung adenocarcinoma.

To screen for immune-related lncRNAs, we calculated Co-expression analysis and generated the co-expression network map between immune-related lncRNAs and mRNAs. The correlation analysis was determined by Pearson's correlation. LncRNAs with correlation coefficient > 0.5 and *P*< 0.05 were used for further analysis. Cytoscape software version 3.6.0 was used to visualize the resulting network. As shown in Figure 2, Blue is immune-related mRNA and red is co-expressed lncRNA.

### Identification of prognostic value of immune-related lncRNAs

In order to find the immune-related lncRNAs related to the prognosis, we annotated the above lncRNAs and clinic information and performed the influence of each lncRNA on prognosis. Prognostic analysis was performed on lncRNAs by univariate Cox regression. The threshold value was set at *P*< 0.05, and 147 immune-related lncRNAs with significant prognostic value were eventually obtained (Supplementary Table 2).

### Construction immune-related lncRNA clusters

**Figure 3:**
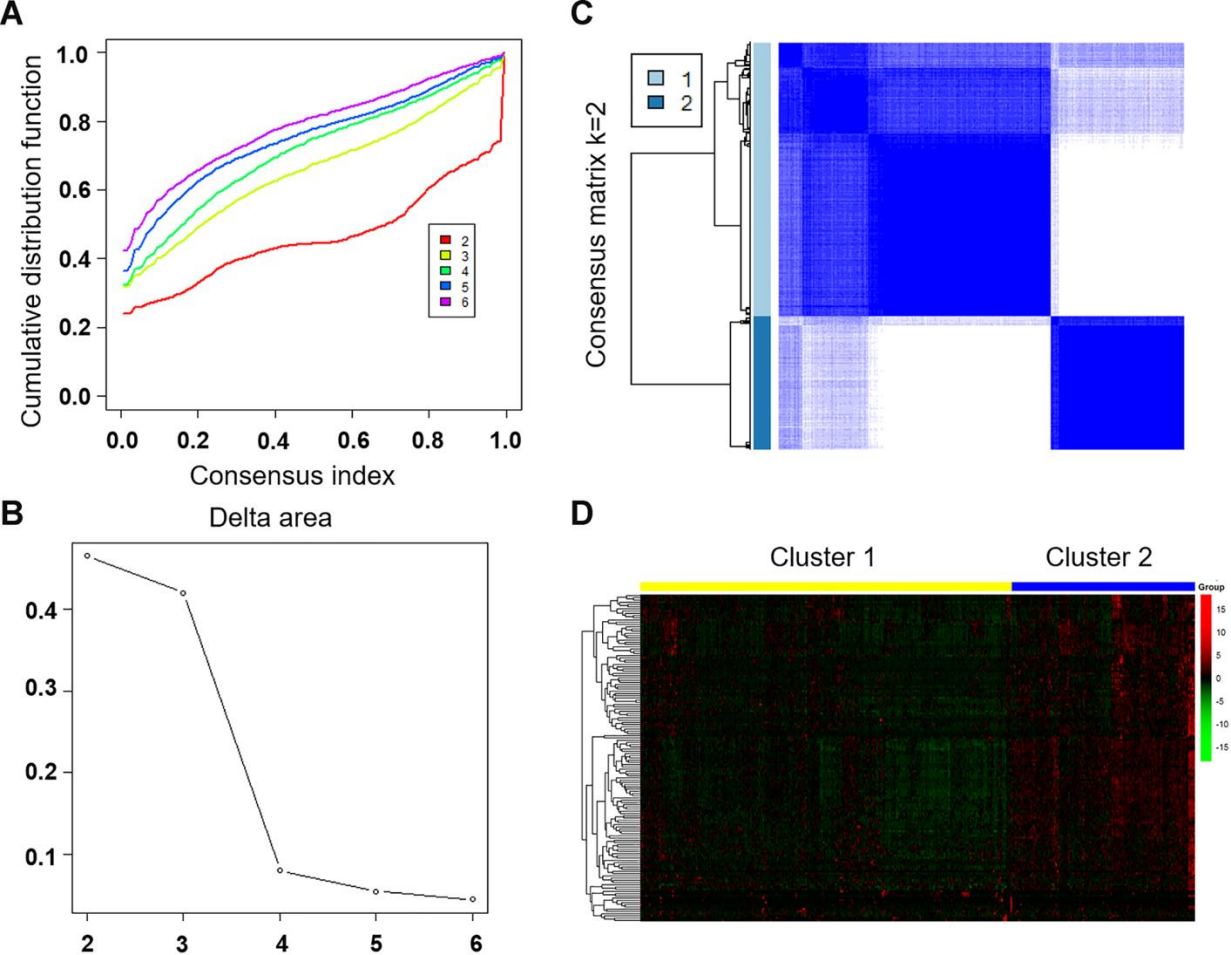
**Unsupervised clustering of LUAD using immune-related lncRNA expression data.** (**A**) Consensus CDF plot and consensus index for k =2 to 6 are represented. X axis represents consensus index, Y axis represents CDF. (**B**) Cumulative distribution function graph of the consistency matrix at K = 2. The blue and white heatmap displays sample consensus. (**C**) Delta area score map. X-axis represents the number of clusters and Y-axis represents the relative increase in cluster stability. (**D**) Heat map represents the expression of immune-related lncRNA in cluster 1 and cluster 2. Red represents high expression and green represents low expression. CDF: Cumulative Distribution Function.

To separate LUAD samples into tumor clusters with different immune phenotypes based on immune-related lncRNA, we mapped 147 immune-related lncRNAs to the expression profile of LUAD samples to perform consistent clustering using CCP tool. The maximum number of clusters was set to 6 (Figure 3A). CCP analysis revealed the most stable results when divided into two tumor clusters, which were named cluster 1 and cluster 2 (Figure 3B, 3C). Figure 3D showed the expression of immune-related lncRNA in two clusters. Red is high expression and green is low expression. Yellow represents cluster 1 and blue represents cluster 2.

### Comparison of composition and immune cells infiltration of TME in different immune-related lncRNA clusters

**Figure 4:**
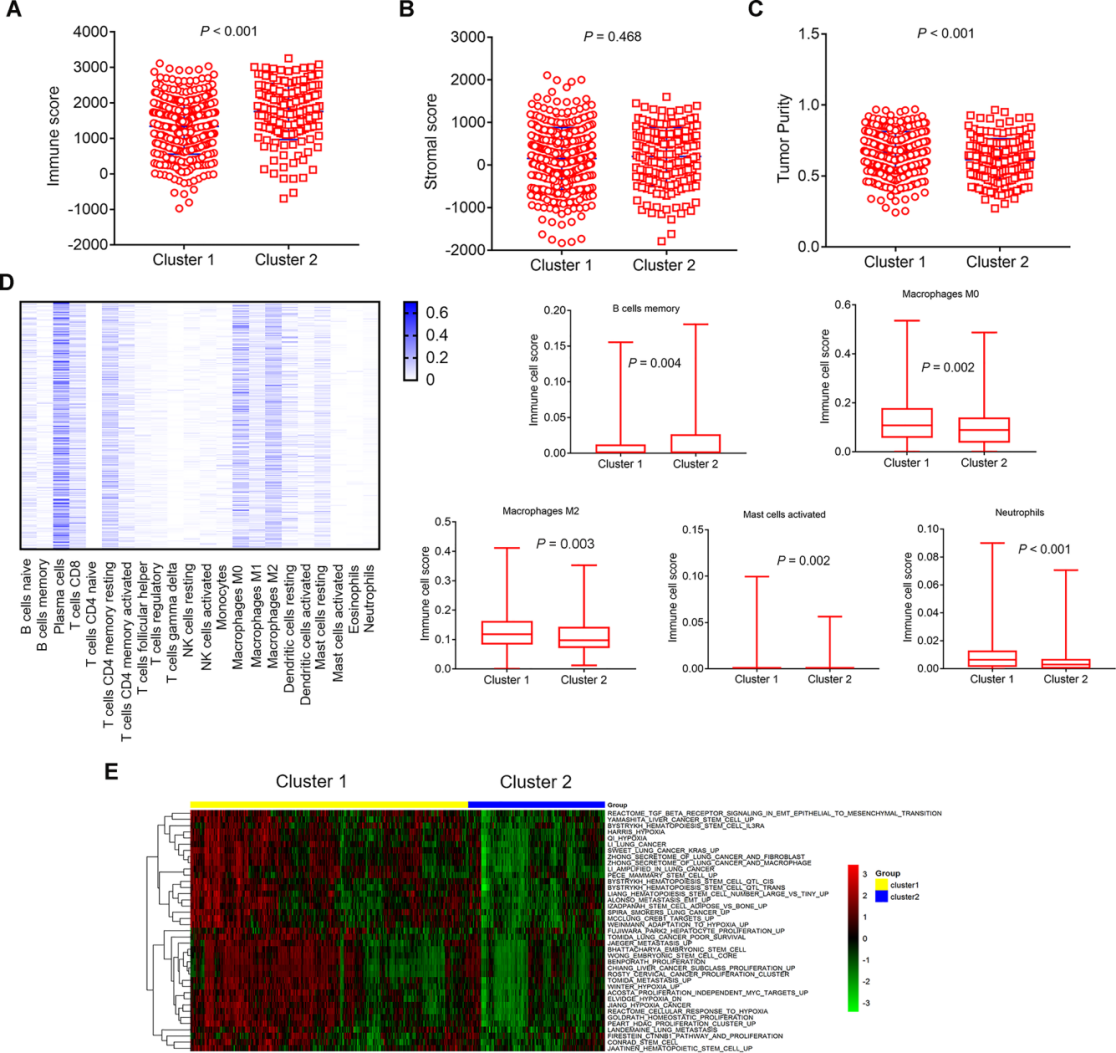
**Comparison of composition and immune cells infiltration of the tumor immune microenvironment in LUAD samples and GSVA pathways analysis with differential enrichment.** (**A**–**C**) Comparison of composition of TME (immune score, stromal score, and tumor purity) between cluster 1 and cluster 2. (**D**, left) Heat map represents the relative levels comparison of different immune cell types. The darker the blue, the higher the expression. (**D**, right) Comparison of immune cells infiltration (Macrophages M0, Macrophages M2, Mast cells activated, Neutrophils, B cells memory). (**E**) Heat map represents the the changes of related pathways in cluster 1 and cluster 2. The color changes from green to red, indicating an increase in the value of the enriched score. Yellow represents cluster 1 and blue represents cluster 2. TME: tumor microenvironment; GSVA: Gene Set Variation Analysis.

ESTIMATE algorithm was used to calculate immune score, stromal score and tumor purity (estimate score) according to the gene expression profiles data of 500 LUAD samples. Then we analyzed the disparities of immune score, stromal score, and tumor purity (estimate score) in different immune-related lncRNA clusters. The cluster 2 had higher immune score, and lower tumor purity than the cluster 1 (Figure 4A, *P*<0.001; Figure 4C, *P*<0.001). Similarly, the cluster 2 also had higher stromal score, although these differences were not statistically significant (Figure 4B, *P*=0.468).

The CIBERSORT method was used to estimate the immune cell composition of 500 LUAD samples and quantify the relative levels of different cell types in a mixed cell population. All results were normalized relative proportions by cell type. Heat map showed the relative levels comparison of different immune cell types in LUAD samples (Figure 4D, left). There were differences in 5 types of immune cells. The relative levels of immune cell types in cluster 1 were higher than the cluster 2, such as Macrophages M0 (*P*=0.002), Macrophages M2 (*P*=0.003), Mast cells activated (*P*=0.002), Neutrophils (*P*<0.001), apart from B cells memory (*P*=0.004), which was lower in cluster 1 (Figure 4D, right).

### Pathways enrichment analysis

We performed GSVA analysis on different genes in different immune-related lncRNA clusters to obtain the changes of related pathways. Heat map showed the change of different samples in cluster 1 and cluster 2 (Figure 4E). The color changes from green to red, indicating an increase in the value of the enriched score. Yellow represents cluster 1 and blue represents cluster 2. Unsupervised clustering of the average gene set score could clearly separate the cluster 1 and cluster 2. GSVA analysis showed that differential pathways were related to stem cell biology and EMT.

### Comparison of somatic mutations associated with immune activation in different immune-related lncRNA clusters

**Figure 5:**
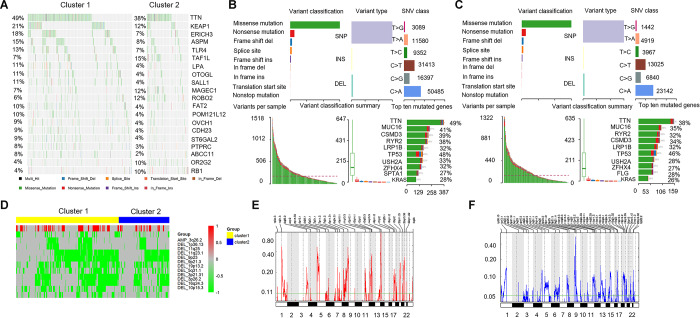
**Comparison of mutant genes associated with immune activation and CNVs profile.** (**A**) Mutation spectrum of the top 20 mutated genes with the highest number in cluster 1 and cluster 2. (**B**, **C**, above) The variant classification, variant type, and SNV class in cluster 1 and cluster 2. (**B**, **C**, below). Variants per sample, variant classification summary, and top ten mutated genes. (**D**) Comparison of CNVs profile (amplification and deletion) in cluster 1 and cluster 2. Yellow represents cluster 1 and blue represents cluster 2. Red represents the amplified portions and green represents deleted portions. (**E**, **F**) Areas with significant copy amplification and deletion of lncRNAs. G-scores (left) are normalized values of the amplification/ deletion signals and indicate the degree of gene amplification/ deletion. The larger the G-scores, the greater the degree of gene amplification/ deletion. The Q value (right) is the significance level of the amplification/ deletion, and the green line represents the threshold value of the significance level with Q value = 0.25. CNVs: copy number variations; SNV: single nucleotide variant.

To identify the disparities of mutated genes in different immune-related lncRNA clusters, we selected the top 20 mutated genes with the highest number from each cluster to generate different mutation spectrum. As shown in Figure 5A, significant disparities could be found in these mutant genes between cluster 1 and cluster 2, such as, KEAP1 (21% vs 12%) and TLR4 (13% vs 7%). Figure 5B showed the mutation characteristics of the genes in cluster 1. The results showed the variant classification, variant type, and SNV class in cluster 1 (Figure 5B, above) and variants per sample, variant classification summary, and top ten mutated genes (Figure 5B, below). Figure 5C showed similar results for cluster 2, but with some differences. The number of each SNV class in cluster 1 were more than cluster 2. The top 10 mutant genes in cluster 1 were TTN(49%), MUC16(41%), CSMD3(39%), RYR2(38%), LRP1B(32%), TP53(48%), USH2A(33%), ZFHX4(32%), SPTA1(27%), and KRAS(28%). While the top 10 mutant genes in cluster 2 were TTN(38%), MUC16(35%), RYR2(32%), CSMD3(34%), LRP1B(32%), TP53(46%), USH2A(28%), ZFHX4(27%), FLG(28%), and KRAS(26%). The top 10 mutation genes and mutation frequencies of cluster 1 and cluster 2 were not significantly different, except for TTN (different frequencies), SPTA1 and FLG (different genes).

### Comparison of CNVs profile in different immune-related lncRNA clusters

We analyzed CNVs data of 497 LUAD samples and extracted the copy number spectrum of lncRNA. As shown in Figure 5D, the disparities of CNV segments between cluster 1 and cluster 2 were identified, and both the amplified and deleted portions were enriched in cluster 1. In addition, we used a GISTIC algorithm to identify frequently changing areas in LUAD genome. Many areas were found with significant copy amplification and deletion of lncRNAs (Figure 5E, Figure 5F).

### Identification of prognostic value of immune-related lncRNA clusters in the training and validation group

**Figure 6:**
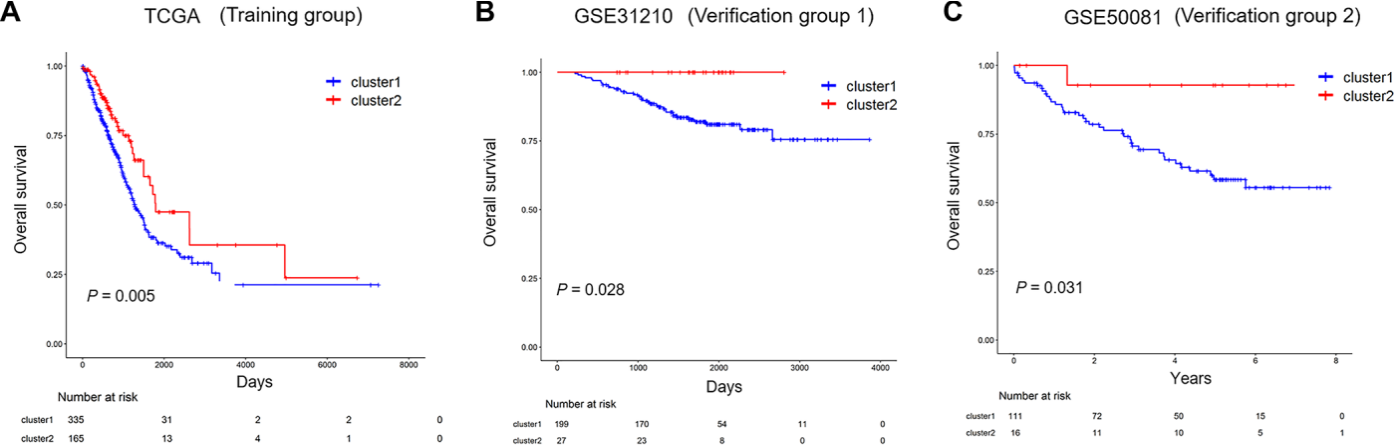
**The prognostic of immune-related lncRNA cluster in the training and validation group.** (**A**) Kaplan–Meier survival curves for overall survival in TCGA (training group). (**B**, **C**) Kaplan–Meier survival curves for overall survival in GSE31210 and GSE50081 (validation group). TCGA: The Cancer Genome Atlas.

To investigate the prognostic of immune-related lncRNA clusters, the dataset from TCGA, including 500 LUAD samples was used as a training group. All patients of training group were classified into cluster 1 and cluster 2 according to immune-related lncRNAs. Kaplan-Meier survival curves showed that cluster 1 has shorter overall survival (OS) than cluster 2 (Figure 6A, *P*=0.005).

Two independent validation datasets GSE31210 and GSE50081 were applied to verify the reliability of the immune-related lncRNA clusters impact on the prognosis of the patients. Kaplan-Meier survival curves based on the immune-related lncRNA cluster were both significantly different in validation datasets GSE31210 and GSE50081 (Figure 6B, *P*=0.028; Figure 6C, *P*=0.031). Similarly, the survival rates for cluster 1 were both lower than that of cluster 2 throughout the follow-up time.

## DISCUSSION

With the development of large-scale sequencing technology and bioinformatics methods, lncRNAs have been revealed to be involved in carcinogenesis and cancer development [13, 29, 32]. Accumulating studies have also demonstrated the significance of lncRNAs in LUAD, including roles as drivers of tumor-suppressive and oncogenic functions, micro-RNA competitors, and diagnostic biomarkers [33–35]. Recently, the involvement of lncRNAs in immune regulation have been widely reported [15–18]. Therefore, immune-related lncRNAs may be used to explore different immune stratification and analyze their mechanism characteristics in LUAD. In our study, we obtained 147 immune-related and prognostic lncRNAs and constructed two immune-related lncRNA clusters group using 500 LUAD samples from TCGA.

Interestingly, we found two clusters of LUAD samples based on immune-related lncRNAs with significant differences in immune microenvironment and genomic alterations. Infiltrating immune cells are an integral component of TME and play an important role in shaping the TME [36, 37]. Previous reports have provided elegant analysis how lncRNAs involve innate and adaptive immune responses by modulating the functional status of immune cells [15–18, 22–24]. We analyzed composition and immune cell types of the TME in different immune-related lncRNA clusters. The results showed that the cluster 1 had lower immune score, and higher tumor purity than the cluster 2. Simultaneously, there were differences in 5 types of immune cells. The relative levels of immune cell types in cluster 1 were higher than the cluster 2, such as Macrophages M0 (*P*=0.002), Macrophages M2 (*P*=0.003), Mast cells activated (*P*=0.002), Neutrophils (*P*<0.001), apart from B cells memory(*P*=0.004), which was lower in cluster 1. The 5 types of immune cells are thought to be involved in the regulation of innate and adaptive immune responses and play an important role in anti-tumor immunity. M2 macrophages play a suppressive role in immune function. Our result showed that the immune score in cluster 1 was low, and the components of most macrophages (M0 and M2) were relatively high, indicating a stronger immunosuppressive response in cluster1. The above results suggested that cluster 1 had a stronger immunosuppressive TME than cluster 2 in LUAD samples.

To further characterize the relationship between immune-related lncRNA clusters and cancer cell phenotypes, genes from the MsigDB gene set related to EMT, stem cells, hypoxia, and proliferation were used [38]. GSVA analysis on the differential genes between cluster 1 and cluster 2 was also performed to obtain the changes of related pathways. The result showed that differential pathways were related to stem cell biology and EMT. The cluster 1 and cluster 2 were clearly separated using the GSVA method. These results revealed that these immune-related lncRNA clusters may be involved in stem cell and EMT functions.

The majority of work to date have confirmed the somatic mutations were an essentiality of carcinogenesis and cancer development [26, 29, 39]. A recent study comprehensively evaluated the properties of lncRNAs from different cancer types in an attempt to to explore the associations between somatic mutations and lncRNA expression [29]. LncRNAs were commonly downregulated and carried low mutation frequencies and non-silent mutations in most cancer types and were determined with several conserved and cancer specific functions. In our study, we identified the characteristics of mutation spectrum in different immune-related lncRNA clusters. The results showed that KEAP1 (21% vs 12%), and TLR4 (13% vs 7%) had significant differences between cluster 1 and cluster 2. Studies had shown that activation of the Nrf2 protein and its regulatory signaling pathways caused by KEAP1 mutations is conducive to the survival of lung cancer cells and resistance to chemotherapy drugs [40]. Toll-like receptors (TLRs) are highly conserved during evolution, are widely expressed in immune cells, and play an important role in triggering inflammatory responses in the innate immune system [41]. Evading immune destruction is an important sign of cancer development. There is a definitive link between chronic inflammation and cancer, where TLRs play an important role in the immune response against tumor cells. Recently, TLRs were found on tumor cells, and their activation may coordinate downstream signaling pathways that play a vital role in tumorigenesis and tumor progression [42]. As a lung cancer cell sensor, TLR4 regulate lung cancer progression in terms of cell growth, invasion, angiogenesis, and tumor stem cell behavior [43]. The above results suggested that there was a difference in the mutation spectrum of cluster 1 and cluster 2, and cluster 1 had a higher mutation frequency than cluster 2, especially the mutant genes, such as KEAP1 and TLR4, that played a key role in the progression of lung cancer.

CNVs can lead to different degrees of differential gene expression, and multiple CNVs in the genome can cause heterogeneity of genomic and molecular phenotypes, leading to the occurrence and development of complex diseases including cancer [27, 28]. We analyzed CNVs data of 497 LUAD and extracted the copy number spectrum of lncRNA. The disparities of CNV segments between cluster 1 and cluster 2 were identified, and both the amplified and deleted portions were enriched in cluster 1. A query revealed that the tumor suppressor gene CDKN2A (cyclin dependent kinase inhibitor 2A) was significantly deleted in cluster 1 in the 9p21.3 region. CDKN2A is frequently inactivated in many malignant tumors, and is closely related to lung cancer progression. The above results suggested that there was a difference in the CNV segments of cluster 1 and cluster 2, and immune-related lncRNA may be associated with the occurrence and development of LUAD.

Finally, we investigated whether immune-related lncRNA clusters could predict the prognosis of the LUAD patients. Immune-related lncRNA clusterization analysis can well divide LUAD patients into cluster 1 and cluster 2 in the training and validating group, and cluster 1 was related to worse prognosis of LUAD patients. The above finding confirmed that the immune-related lncRNA cluster had reliable prognostic value.

In conclusion, we constructed two immune-related and prognostic lncRNA clusters with significant differences in immune microenvironment and genomic alterations in LUAD samples. The immune-related lncRNA clusterization analysis of LUAD samples provided a new insight into the stratification of patients with different immune phenotypes as well as in-depth understanding of immune molecular mechanisms in LUAD. This study also has certain limitations. Firstly, this study only analyzed the immune-related lncRNA and genetic data in LUAD samples, but not the normal paired lung tissue. Secondly, this study is a bioinformatic and retrospective research. Due to lack or inconsistency of clinical data, multivariate regression analyses of immune-related clusters cannot be performed uniformly.

## MATERIALS AND METHODS

### Data source and processing

All RNA sequencing (RNA-seq) profiling data, including 500 LUAD samples were downloaded from The Cancer Genome Atlas (TCGA, https://tcga-data.nci.nih.gov/tcga/;LUAD). All the clinical information related to these samples was also obtained. Two independent validation datasets GSE31210 and GSE50081 were downloaded from GEO (Gene Expression Omnibus) database (https://www.ncbi.nlm.nih.gov/geo/). In total, 226 lung samples were included in the GES31210 dataset, and 127 lung samples were included in the GES50081 dataset.

The list of immunoregulatory genes were downloaded from the immune-related ImmPort database (https://immport.niaid.nih.gov). We also download lncRNAs data from the Ensembl database (https://asia.ensembl.org/index.html). Since the GEO database contains data from the u133plus2.0 platform, the probe is re-annotated to the Ensembl database, and the data of lncRNAs is filtered out by the annotation of lncRNAs. The study was approved by the ethics committee of the Shandong Cancer Hospital.

### Unsupervised clustering analysis

To analyze the expression of immune-related lncRNA in different tumor clusters, the R software package of Consensus Cluster Plus (CCP) [44] was applied to perform a tumor cluster classification. The algorithm began by subsampling a proportion of items and a proportion of features from a data matrix. Each subsample was then partitioned into up to k groups by a user-specified clustering algorithm. The maximum number of clusters was set to 6, and the optimal number of clusters was determined according to the consensus index and Cumulative Distribution Function (CDF). Heat map clustering of the immune-related lncRNAs were drawn using the R software plots package.

### ESTIMATE analysis

An ‘Estimation of Stromal and Immune cells in Malignant Tumours using Expression data’ (ESTIMATE) algorithm is applicated to infer the levels of infiltrating stromal and immune cells and estimate tumour purity in tumour samples using gene expression data [45]. The algorithm is publicly available through the SourceForge software repository (https://sourceforge.net/projects/estimateproject/). The algorithm is based on single-sample gene set enrichment analysis and generates three scores: 1) stromal score (the presence of matrix in tumor tissue); 2) immune score (the infiltration of immune cells in tumor tissue); 3) estimate score (the inference of tumor purity).

### CIBERSORT analysis

To estimate the immune cell composition in the sample, the analytical platform CIBERSORT (https://cibersort.stanford.edu/) is used to quantify the relative levels of distinct immune cell types within a complex gene expression mixture [46]. CIBERSORT's deconvolution of gene expression data provides valuable information about the composition of immune cells in a sample.

### Gene set variation analysis (GSVA)

GSVA, a pathway enrichment method, was used to estimate variation of pathway activity over a sample population. The R software package of GSVA was downloaded at http://www.bioconductor.org [47]. The prediction of the pathway under different disease states was made by the signal value of the gene and the pathway in which the gene was located. The enriched score value of each sample was predicted by the signal value of the gene, and the pathway with differential enrichment in the two groups was obtained. The screening standard *P*<0.05, and the FDR<0.05.

### Somatic mutations analysis

All gene somatic mutations of 500 samples were also downloaded from the TCGA. Fisher's exact test was used to identify differential genes with different immunotypes (*P* <0.05), and the mutated genes with the highest number from different clusters were compared to generate different mutation spectrum. Moreover, we also showed the variant classification, variant type, and SNV class in each cluster. Similarly, variants per sample, variant classification summary, and top ten mutated genes were all analyzed.

### CNVs analysis

The CNVs of all samples from TCGA LUAD were downloaded. Genomic Identification of Significant Targets in Cancer (GISTIC) [48] was used to visualize regions in the genome to show amplification and deletion in the samples. The CNV data of 497 LUAD samples downloaded from TCGA using GISTIC 2.0 software was analyzed and the CNV spectrum of lncRNA was extracted. Taking the number of copies greater than 1 as the threshold of copy amplification and less than -1 as the threshold of copy deletion, we calculated the ratio of copy amplification and deletion for each lncRNA. Finally, we identified the disparities of CNV segments in different immune-related lncRNA clusters using Fisher’s exact test.

### Statistical analysis

Using survival package of R language, survival analysis was used to compare the survival curves of different immune-related lncRNA clusters. The Cox proportional hazard regression analyses was performed prognostic analysis. The correlation analysis was determined by Pearson's correlation. In addition, t-test, Pearson's Chi-square test or Fisher’s exact probability test was used to estimated statistical significance. *P* < 0.05 was considered statistically significant.

## Supplementary Material

Supplementary Table 1

Supplementary Table 2
